# Talks with Dr. Mandeville

**Published:** 1892-07

**Authors:** 


### HALL'S JOURNAL OF HEALTH

TRUTH DEMANDS NO SACRIFICE; ERROR CAN MAKE NONE.

Vol. 39. JULY, 1892. No. 7.

### TALKS WITH DR. MANDEVILLE.

No. 6.

Doctor, is it convenient for you to resume your remarks on the nervous system ?

Answer. — As you please, I am at your service for a couple of hours. To proceed :

There is a difference between a telegraph message and one delivered by the nerves. The message which is sent out from the central telegraph office is exactly the one which is received. This is not the case with the nerves. When the hot spoon touches my hand, a message goes up the spinal cord and returns, but the message does not excite the nerves which connect with my hand, but excites certain muscles which make my hand jump, and this is a different thing from the message itself. How is it produced ? We must suppose that there are two central offices ; any message which we can actually transmit in the body is really a very complex one. It is started upon a great number of the ends of the small nerve filaments, and each of these carries its message quite separately into the large cords.

These nerve filaments are separated in the cords by a fatty matter which insulates them, and the complicated message arrives without any disturbance or loss of any of its parts, into "grey matter" (typified by the collection of card houses) of the nervous system, composed of a number of cells or corpuscles, that is to say, little round substances which have a skin and a nucleus ; they are just like nerve substance itself, and capable of falling into easier positions whenever disturbed. They are even more unstable than the matter of the nervous fibre.

The collection of messages arrives in the central office, and constitute a single message. For example : If you send a message by telegraph you have different motions for the different letters which are indicated by the combination of two signals, either of which can turn to the right or left. But a nerve can only carry one kind of a message—it can tell at the end if it has been disturbed at the beginning. In order to carry a complex message, therefore, such as "A hot spoon has been laid upon my hand," a great many nerves have to be employed. You can see that by a complication of signals it would be possible to convey a message like the one specified, one thread can only say "I have been disturbed," but a number would say "We have all been disturbed," and when this compound message comes into the office, what the grey matter does is to combine all the parts.

That process is called co-ordination, which means keeping company in order. The several parts of the message being united and sent on as a single message to the next central office. This next central office does another thing—the one message which is sent to it is "Pull a certain muscle or number of muscles ;" but in order to keep those muscles in order it has got to send out a large number of messages, each of which goes to a particular place in each particular muscle ; and it un-co-ordinates the message which has just been co-ordinated at the previous central office so as to produce this motion of the muscle.

Now, if you feel down your back you will find a number of knobs ; what you feel there is the spine, which is made up of a great number of different bones, and each of those small bones has a hole in it, so that when they are put one upon another, it is like a lot of pill boxes, so placed, each of which has a hole in the top and bottom of it, so that you can put a thread through all of them. The thread which goes through those holes is called the spinal marrow. It is made up of white nerve threads and fatty matter. When any message is sent up from my foot, as when you tread on my toe, that message goes in at the back between those bones into the spinal cord, the nerve threads there end in a certain mass of grey matter, a "knot" it is called or "ganglion." Close to that there is another ganglion from which messages go out from a hole in front between the same bones, and those messages which come down from the front go to my toe and tell it to get out of the way.

The office of those two sets of ganglions in your spinal cord is that one co-ordinates a number of sensory messages coming in from behind and transfers them, so co-ordinated, to the other knot, which sends the corresponding messages to a number of muscles, telling them to move in the right way. Thus, you see that every pair of nerves in the spinal cord may be regarded as a small brain, because what our brain does upon the whole is to receive messages and send out the answers, teaching each part how to move. We may in the first place, therefore, regard our brain as a number of small cords which go all the way down our back.

[To be continued.]

